# Exploring the feasibility and acceptability of community paramedicine programs in achieving vaccination equity: a qualitative study

**DOI:** 10.1186/s12913-024-11422-0

**Published:** 2024-09-04

**Authors:** Monica L. Kasting, Alfu Laily, Sidney J. Smith, Sathveka Sembian, Katharine J. Head, Bukola Usidame, Gregory D. Zimet, Laura M. Schwab-Reese

**Affiliations:** 1 Department of Public Health, Purdue University, 812 W. State Street, West Lafayette, IN 47907 USA; 2 Cancer Prevention and Control Program, Indiana University Simon Comprehensive Cancer Center, Indianapolis, IN USA; 3 Wheldon School of Biomedical Engineering, Purdue University, West Lafayette, IN USA; 4 Department of Communication Studies, Indiana University, Indianapolis, IN USA; 5 Emeritus, Department of Pediatrics, Indiana University School of Medicine, Indianapolis, IN USA

**Keywords:** Health inequities, Vaccination Coverage, Rural Population, Paramedicine, Community Paramedicine

## Abstract

**Background:**

Mobile Integrated Health-Community Paramedicine (MIH-CP) is a novel approach that may reduce the rural-urban disparity in vaccination uptake in the United States. MIH-CP providers, as physician extenders, offer clinical follow-up and wrap-around services in homes and communities, uniquely positioning them as trusted messengers and vaccine providers. This study explores stakeholder perspectives on feasibility and acceptability of community paramedicine vaccination programs.

**Methods:**

We conducted semi-structured qualitative interviews with leaders of paramedicine agencies with MIH-CP, without MIH-CP, and state/regional leaders in Indiana. Interviews were audio recorded, transcribed verbatim, and analyzed using content analysis.

**Results:**

We interviewed 24 individuals who represented EMS organizations with MIH-CP programs (MIH-CP; *n* = 10), EMS organizations without MIH-CP programs (non-MIH-CP; *n* = 9), and state/regional administrators (SRA; *n* = 5). Overall, the sample included professionals with an average of 19.6 years in the field (range: 1–42 years). Approximately 75% (*n* = 14) were male, and all identified as non-Hispanic white. MIH-CPs reported they initiated a vaccine program to reach underserved areas, operating as a health department extension. Some MIH-CPs integrated existing services, such as food banks, with vaccine clinics, while other MIH-CPs focused on providing vaccinations as standalone initiatives. Key barriers to vaccination program initiation included funding and vaccinations being a low priority for MIH-CP programs. However, participants reported support for vaccine programs, particularly as they provided an opportunity to alleviate health disparities and improve community health. MIH-CPs reported low vaccine hesitancy in the community when community paramedics administered vaccines. Non-CP agencies expressed interest in launching vaccine programs if there is clear guidance, sustainable funding, and adequate personnel.

**Conclusions:**

Our study provides important context on the feasibility and acceptability of implementing an MIH-CP program. Findings offer valuable insights into reducing health disparities seen in vaccine uptake through community paramedics, a novel and innovative approach to reduce health disparities in rural communities.

**Supplementary Information:**

The online version contains supplementary material available at 10.1186/s12913-024-11422-0.

## Introduction

Mobile integrated health-community paramedicine (MIH-CP) is a rapidly evolving patient-centered healthcare delivery model within the domain of emergency medical services (EMS) [1, 2]. Community Paramedics (CP)s, a large portion of the MIH-CP workforce, expand the traditional role of EMS personnel to be physician extenders, delivering non-urgent but key medical services such as vaccinations. This is particularly important considering the existing vaccination inequities.

The COVID-19 pandemic highlighted these systemic health inequities, exacerbated existing health disparities, and broadened the gap in access to care. For example, many healthcare visits during the COVID-19 pandemic took place virtually using telemedicine and research shows that low socioeconomic status (SES), rural, and minority populations received less access to telehealth during the pandemic [3, 4]. Furthermore, a study of Rural Health Clinics (RHCs) found that the clinics reported high levels of financial concerns and challenges obtaining personal protective equipment, resulting in them providing fewer preventive services during the pandemic [5].

Vaccination rates also dropped during the pandemic, with early reports suggesting some childhood vaccination rates dropping by as much as 70% in the beginning of the pandemic [6, 7]. These reductions in vaccine uptake are multifactorial and are associated not only with lack of access to care, but also higher levels of mistrust in the medical system and medical establishment among underrepresented minorities as well as people living in rural areas [7]. A potential solution to address these disparities is through trusted messengers, who have the opportunity to change previously held beliefs and increase awareness and acceptability of vaccinations [8]. One example of a trusted messenger is a CP.

CPs are evolving to be a blend of community health workers, social workers, and non-emergency health care providers [1, 2, 9]. Approximately 18 states in the United States (U.S.) have CPs, but the roles vary in scope, training, and authority [1, 10]. Studies have shown that they are positively accepted and reviewed across the quadruple aim framework used to assess the effectiveness of a health care system (i.e., improved patient satisfaction, improved provider satisfaction, reducing healthcare costs, and improved population health outcomes) [1, 11]. While there are limited studies encompassing all of the quadruple aims, review papers have shown that MIH-CP programs are generally perceived positively as a means of bridging the healthcare delivery gap, especially within communities with healthcare shortages, such as rural areas, and can potentially reduce existing disparities [1, 2, 9, 10, 12–15].

This positive reception and patient satisfaction suggests MIH-CP may be a novel approach to address health disparities and improve uptake of preventive health services, including vaccinations [1, 14, 15]. MIH-CP programs are often able to administer vaccines [16–22], but there are few studies specifically examining the impact of this service. Currently, Indiana has more than a dozen MIH-CP programs [23], including many that provide vaccination services. More research is needed to understand program effectiveness and the potential usefulness in improving health equity through these programs. In addition, it is unclear whether community paramedics are receptive to including vaccine administration in their scope of care, which may cause implementation challenges. Therefore, the aim of this study was to determine perceived barriers, facilitators, attitudes, and beliefs of relevant stakeholders (i.e., MIH-CP/EMS providers, leaders, and administrators) regarding implementation of MIH-CP-based adult vaccination services in the state of Indiana.

## Methods

This study was reviewed and approved as exempt by the Institutional Review Boards at both Purdue University and Indiana University.

### Interviews

#### Setting, design, sample, and recruitment

We conducted one-time interviews with three groups of participants: leaders of paramedicine agencies with registered MIH-CP programs (10 interviews), leaders of paramedicine agencies without MIH-CP programs (9 interviews), and state/regional administrators (SRA; 5 interviews). Below we describe how each group was identified and recruited.

The Indiana Department of Homeland Security (DHS), which oversees EMS in Indiana, provided a list of registered MIH-CP programs as of January 2023. Team members contacted the administrators of these programs via email or phone to confirm whether the MIH-CP program was active. Of the 16 registered agencies with an active MIH-CP program, 10 (63%) completed interviews.

To identify non-MIH-CP providers, we used targeted recruitment and identified counties that were demographically similar to the counties served by the MIH-CP interviewees, specifically focusing on rurality (within 4% rurality of MIH-CP interviewees) and average resident age (mean age within 2 years of MIH-CP interviewees). Then, we identified the hospital-based, governmental, paid fire, and private paramedicine agencies in those counties from a registry of ambulance service providers from DHS. We excluded volunteer organizations, as they are quite different in scope and function than organizations with employees. Of the 18 programs identified and contacted, 9 (50%) completed an interview.

Finally, we contacted the state MIH-CP administrators and regional EMS administrators, based on the contact information provided on the DHS website [24]. Approximately half (i.e., 5 of 9) of the state and reginal administrators contacted by the team completed an interview.

### Procedures

All interviews were conducted by study team members (MLK, AL, SJS) trained in qualitative interviewing and were recorded via Zoom. One interviewer (MLK) is a faculty member with a PhD and two are graduate students (AL, SJS). All interviewers are female. No one else was present in the interviews besides the participants and research team members. The interview guides are included as Additional File 1 (leaders of paramedicine agencies) and Additional File 2 (state/regional administrators). The audio files were transcribed in three rounds, one by artificial-intelligence transcriber through happyscribe.com, then verified by two rounds of manual transcriptions carried out by team members with training in qualitative methods (AL, SJS, SS). Following the interview, participants completed an anonymous survey about their demographic characteristics and beliefs about the COVID-19 vaccine. Survey items were adapted from previously validated surveys [25–27], where applicable. The survey codebook is included as Additional File 3. All participants were offered a $50 gift card in appreciation of their time, although many declined due to agency restrictions on accepting gifts.

Interviews started with introductory questions about the participants’ roles within their agency to build rapport, better understand the participants’ experience in EMS, and describe the goals of the research project. The rest of the interview questions focused on MIH-CP program history and functions. However, the questions were tailored to the participants’ experiences with MIH-CP and whether they had vaccination programs. For MIH-CP interviews, the subsequent questions focused on their overall MIH-CP programs as well as their vaccination programs, emphasizing how the programs started, barriers to implementation, operational barriers, and lessons learned. Non-MIH-CP interviews emphasized similar topics except that the questions were framed around their opinions and perspectives on MIH-CP as someone without a program. State/regional administrator interviews focused on higher-level administration of MIH-CP programs.

### Data analysis

We used qualitative content analysis, as described by Schreier, to analyze the transcripts [28]. First, two authors (LMSR, AL) completed an exhaustive and comprehensive review of the transcripts to ensure a thorough understanding of all the data. During these reviews, they took notes on content that was repeated across interviews and areas that were unique to each interview. After gaining familiarity with the material, each author reviewed the transcripts for a second time, specifically focusing on content that was not noted in the first review. Then, each author organized their notes into a first draft of a codebook. This approach is most similar to the codebook development strategy described by Schrier as summarization. As part of our note-taking process, we paraphrased relevant passages. As we developed the codebook, we deleted paraphrases that were superfluous and combined related paraphrases. Then, we used the paraphrases to generate the main category and subcategory names. Although we did not generate the main categories prior to codebook development, our draft codebooks were closely aligned with our objectives because the semi-structured interview guide used to collect data was aligned with our objectives.

Based on these initial drafts, two members of the research team (LMSR, MLK) reviewed the draft codebooks, combined the codebook drafts into a single comprehensive codebook (Additional File 4), and pilot-coded a transcript together. Then, one member of the team (LMSR) applied the codebook to the transcripts. Finally, two members of the team (LMSR, MLK) met to review the coded materials and assess for disagreement in the code application. However, the codebook is quite straightforward and descriptive, so there were no disagreements.

Saturation has multiple meanings in qualitative methods. In qualitative content analysis, as described by Schreier, saturation occurs when each subcategory has at least one code segment (i.e., no subcategory is ‘empty’). Because we used a data-driven approach to develop our codebook, we automatically met the criterion of saturation. That is, if the content was not present in the data, it was not present in our coding framework. Data analyses were conducted using MAXQDA.

## Results

### Sample characteristics

We interviewed 24 individuals who represented EMS organizations with MIH-CP programs (MIH-CP; *n* = 10), EMS organizations without MIH-CP programs (non-MIH-CP; *n* = 9), and state/regional administrators (SRA; *n* = 5). Interviews lasted an average of 41 min (range: 14–75 min). Of the 24 interviewees, 19 responded to the survey provided at the end of the interview. Overall, the sample included highly experienced EMS professionals with an average of 19.6 years in the field (range: 1–42 years). Approximately 75% (*n* = 14) of respondents were male, and all identified as non-Hispanic white. Nearly two-thirds of respondents were fully vaccinated for COVID-19 and had received at least one booster shot (*n* = 12). Another quarter were fully vaccinated without a booster shot (*n* = 5). One respondent received one dose of the COVID-19 vaccine, and one was not vaccinated.

When asked if their programs ever distributed vaccines, more than 75% of agencies reported doing so. This was significantly different between MIH-CP (10 out of 10 distributed vaccines) and non-MIH-CP (5 out of 9 distributed vaccines) programs (*p* < 0.05). Most programs (*n* = 11) discussed distributing COVID-19 vaccines during the pandemic. Flu vaccines were the second most commonly administered vaccine (*n* = 7). Other vaccines included Tetanus, Hepatitis A, and childhood vaccines. Some agencies partnered with other organizations (i.e., primary care providers, health departments, and schools) and were willing to give any vaccines requested by these partners. These partnerships and structures are further discussed in the next section.

### Vaccine program structure and organization

All vaccine programs fit into one of three structures: outreach for a separate agency, extension of existing MIH-CP services, or standalone programs focused on vaccine distribution.

#### Outreach for Separate Agencies

Most vaccine programs were outreach for a separate agency, generally the county health department during the COVID-19 pandemic. In Indiana, many county health departments sponsored mass vaccine clinics and/or provided in-home vaccines for individuals unable to leave their homes. EMS agencies provided staffing for both approaches. One individual shared that during the COVID-19 mass clinics “*the state said that anywhere they were administering vaccines*,* they had to have a paramedic on site.”* (Non-MIH-CP-15). Some agencies allowed their staff to go during normal work hours, while others treated it as volunteer/non-work time. Generally, the MIH-CP programs were more focused on providing in-home services, although a couple of non-MIH-CP programs also provided these services. As one participant explained, “*Let’s do what paramedicine’s meant to do*,* and it’s to be mobile…*” (MIH-CP-08). Generally, these programs followed the same administrative processes:

“So basically, county health nurse will identify Mrs. Smith at 1234 North Main Street, needs a vaccine. Can you do it on this date? Sure, we’ll do it. We’ll send all the information back to the county health nurse and then she’ll enter it in [the state vaccine registry]. And that’s kind of the partnership we have is we’re the boots on the ground and they’re the paperwork side of things, which is obviously the least fun part.” (MIH-CP-05).

At least one program continued partnering with the county health department beyond the COVID-19 vaccine clinics, including providing vaccines to students in schools and routine vaccines in people’s homes. These arrangements had several benefits for EMS agencies: reduced administrative burden, financial compensation, and relationship building with community organizations. As discussed above, the health department was responsible for procuring and storing the vaccines, managing the schedule, and documenting the distribution with the state vaccine registry. This administrative oversight was particularly helpful when the storage and maintenance of multiple COVID-19 vaccines became complicated. As one participant explained:

“It got crazy. Like you had to order your patients in such a way to where your vaccines weren’t expiring. So, we had a fridge inside of the vehicle, but it does not get to cold storage temperatures. So, it’s only maintaining. So yeah, you had to schedule your Johnson and Johnson’s first and then your Modernas and then your Pfizers…” (MIH-CP-10).

Providing vaccines as an extension of the health department was also financially beneficial for some agencies. All agencies were eligible for reimbursement for vaccine administration as part of a state-wide program. One individual explained, “*We got compensated for all those. I think we got like seventy-five dollars- seventy five to one hundred dollars for- per dose.”* (MIH-CP-04). However, some agencies preferred to use the opportunity to build relationships. One individual described their motivation as “*just to help the health department.”* (MIH-CP-09). For many agencies, these programs ended when the mass COVID-19 vaccine clinics ended. Some, including non-MIH-CP programs, used the existing processes and relationships as an opportunity to continue the partnerships, including “*a vaccine clinic at our school.”* (Non-MIH-CP-12).

#### Extension of Current Services

Some MIH-CP programs also provided vaccines as an extension of their current services. Several programs offered vaccines to all existing MIH-CP patients. A primary care provider or health department was responsible for vaccine storage and documentation in these instances. Other extensions reflected the uniqueness of the MIH-CP programs. For example, one MIH-CP program operated out of a community center that hosted a weekly food bank. As demand for the food bank increased, MIH-CP personnel decided to pilot a vaccine clinic, which became the basis for mass vaccine drive-through clinics in the state:

“We tied it into the food distribution. So, people were already here, they were already in line. They would get their food, and as they drove through, we flagged the ones that would like- you know, they said, ‘yeah, we’ll do a flu shot as well.’ It was a simple- it started off with a post-it note on their windshield. And as they came through the food distribution, we’d flag them into the other part of the parking lot, and they would stay in their car, roll down their window, we would vaccinate them, and then we’d move them off just to the side to stay there for their 15 minutes to make sure that they weren’t having a reaction. Their instructions were, if you start feeling funny or ill in any way, honk your horn, turn on your flashers, we’ll be right there.” (MIH-CP-06).

Another MIH-CP program was integrated into an occupational health program and had provided vaccines to their patients since 2013. Generally, this consisted of on-site vaccine clinics, particularly for employers who mandated the vaccines. For other employers, program staff “*just made ourselves available.*” (MIH-CP-07). During the pandemic, this program expanded its vaccine services to other MIH-CP programs. For example, they regularly held clinics at Salvation Army and transitional housing centers. During these events, they started “*providing vaccines at every single one of those community events. And that was just simple walk up.”* (MIH-CP-07).

Because these programs were unique, the relative benefits and challenges were also unique. Some agencies acted as independent vaccine providers, while others’ administrative structure was more similar to that of the agencies acting as outreach (i.e., purchasing, storage, and documentation were managed by a separate agency). Agencies acting as independent vaccine providers did not frame purchasing, storage, or documentation as challenging. However, these agencies had a history of vaccine administration before the COVID-19 pandemic, meaning they had built sufficient infrastructure (e.g., staff, space, and financial resources) for their day-to-day operations.

#### Standalone Program

Only one MIH-CP program had a standalone program focused exclusively on vaccines, which started in 2020. The goal was to provide vaccines in schools for staff and students, with a particular emphasis on vaccines required to attend school. During the pandemic, the program shifted to “*a lot more work with COVID vaccines and testing*” (MIH-CP-08). After schools began reopening, the team learned that a local hospital had started providing a traveling nurse to schools to provide vaccines, which duplicated their service. They decided to shift the focus to “*really just finding those gaps and needs.”* (MIH-CP-08). For this community, that looked like:

“Let’s do what paramedicine’s meant to do, and it’s to be mobile, right, to go out and fill that fill that gap. So if we have students that are getting to that point where school is going to kick them out because they haven’t met their mandated vaccines, we’ll go out and do that. We’ll put clinics together and fill that piece….We have some vaccinations- for HPV and meningitis I believe - that we needed to- we knew that was the right age, so we connected with the school nurse there and did clinics for the [the local college] students.” (MIH-CP-08).

For this program, vaccine storage and documentation were not reported as challenges. The primary challenge was finding the right partnerships and gaps, although there were also financial challenges. Because they operated as a standalone program, they also managed purchasing the vaccines. The administrator described one related issue as, “*You have to be very strategic about it. And we run into that. You know*,* there are a few where we’ve had some expire because we haven’t got shots in arms and you eat that cost.*” (MIH-CP-08).

### Vaccine program challenges

When talking about challenges related to providing vaccines through an MIH-CP program, participants reported a range of challenges, including concerns about funding, vaccine hesitancy in communities, and vaccines as a low priority for MIH-CP.

#### Funding

One participant described the funding issue as “*Vaccines aren’t sexy. It’s not a big money maker. It’s just- It’s one of those things that has to be done*.” (MIH-CP-08). During the COVID-19 pandemic, the state had a program for reimbursing vaccines. Since that program expired, there has been no funding for vaccine distribution through MIH-CP. Without this funding, the biggest barrier for many agencies was “*really just having the money to cover the supplies and the uh cost of actually getting the money out there to do it.”* (MIH-CP-01). Even if funding was available, the administrative burden can be overwhelming. One participant described their program’s decision to stop providing vaccines as:

“But there’s just too much already on a day-to-day basis to where even just that minor ask that they’re trying to ask for it’s becoming too burdensome….it would be fantastic if everyone in our organization also had a secretary, right? I mean, that would be- just someone to help. I’m talking about interns or whatever….you’re sacrificing a lot of your personal time in order to do that, because it’s just not- the reimbursement is just not there to really build up the workforce how it needs to be.” (MIH-CP-10).

The lack of established funding mechanisms was perceived by some participant as evidence that it was not a high priority for the state. As one participant said, “*I’m here to serve my community. So*,* I don’t mind going out there and helping somebody and administering that. But if that was something the health care field thought we should do all the time*,* then there have to be some kind of funding mechanism for that.*” (Non-MIH-CP-16).

In discussing funding challenges, a few other participants discussed how the lack of MIH-CP infrastructure and state policies impeded reimbursement and billing mechanisms. A state/regional administrator explained that many state agencies oversee vaccine regulations. The “*Department of Health*,* because they regulate vaccines. They have reimbursed for some of it”*, “*the FFSA* [Family and Social Services Administration] *was covering* [MIH-CP vaccines] *for Medicaid”* and the Department of Homeland Security play roles in who is allowed to administer vaccines and reimbursement (SRA-21). Some MIH-CP administrators believed that the lack of policies governing MIH-CP contributed to the limited reimbursement opportunities. One participant said,

“We just don’t have a standard documented [reimbursement policy] in the state of Indiana….I mean, there are other states that have it out there. I think Minnesota is a prime example, but yeah. What does that look like for the state of Indiana? And let’s get it written into policy, and it’s been talked about for the last several years, and it’s supposed to be coming up, but it’s just nature of how that works.” (MIH-CP-08).

#### Vaccine Hesitancy

Because most of the programs provided vaccines to individuals who requested them, vaccine hesitancy was not a primary challenge. As one participant described, “*I mean*,* because we’re not beating their door down and jabbing them without their permission*,* right? So if we’re there for a service that they’ve requested*,* uh*,* I don’t see there being any divide. Uh*,* I don’t see there being any issue.”* (Non-MIH-CP-15). However, many of the participants described wide-spread vaccine hesitancy in their communities. One explained that, “*Yeah*,* there’s always hesitant*,* not because we’re doing it. The hesitancy exists because of the vaccine*,* the misinformation from the vaccines. Um*,* when the vaccines became a political issue and a political fireball to use*,* that created a hesitancy*.*”* (Non-MIH-CP-13).

Some people saw addressing vaccine hesitancy as within the scope of paramedicine. Many felt “*comfortable communicating with people”* about vaccines (MIH-CP-01). One went further and said that to address vaccine hesitancy, “*I mean*,* what do you do? You know*,* you can talk to individuals.*” (MIH-CP-06). Others wanted to avoid the “*political involvement*” with vaccines (Non-MIH-CP-18). One participant further explained that “*We see them when they’re sick*,* whether they’re vaccinated or not with COVID or whatever…If they don’t want it*,* they don’t want it. We as an agency*,* don’t push that to outside people.” (Non-MIH-CP-13).*

These differences in opinions may be related to participants’ own feelings about vaccines. In the open-ended question at the end of the post-interview survey, one participant said that,

“Combating misinformation has been required in our vaccination program. Not only with patients but with healthcare providers. More information to healthcare workers delivered in a manner they will digest such as 1-to-2-minute videos would be beneficial. So much information was given, but ultimately ignored during COVID, and I believe the delivery of the information could have been improved. Asking how do we get all our Healthcare providers speaking comfortably, confidently and competently while delivering the same talking points I believe will be critical to build public trust.” (Post-Interview Survey, anonymous).

This view was also shared by another participant who said, “*And even amongst healthcare workers*,* the number of them that just outright refuse for whatever reason is pretty*,* pretty impressive.*” (Non-MIH-CP-17). This division was evident in our post-interview survey questions about vaccine hesitancy. We asked participants how strongly they agreed or disagreed with 12 statements describing vaccine hesitancy like, “Getting a COVID-19 vaccine is a good way to protect me from coronavirus disease.” and “I think COVID-19 vaccines might cause lasting health problems for me.” For all questions, there were individuals who answered “Strongly Agree” and individuals who answered “Strongly Disagree,” respectively. The overall mean score of the 12 items on a scale of 1–5 (with higher numbers indicating more confidence in vaccines) was 3.6 out of 5. However, the anonymous nature of the study precluded us from connecting their interview data with their survey responses.

#### Vaccines as a Low Priority for MIH-CP

A few participants with MIH-CP programs thought vaccines could be a component of their services, but the other services were more critical: “*We were very protective of our Medics because they see only chronic disease patients*,* right - the highest risk patients…So we didn’t- we don’t really do*,* we’re not high-volume vaccines comparatively to some of our other peer programs*.” (MIH-CP-02). Although this view was less commonly described in the interviews, several voiced it in the post-interview survey. One said:

“We should be asking if this is the best way to utilize community paramedics. There are much more beneficial tasks (fall prevention, home modification, collection of health information in case of emergency, risk mitigation) that should be prioritized over vaccines. The vaccinations could be a portion of a holistic health picture but is relatively low priority when it comes to the numbers and severity of those impacted.” (Post-Interview Survey, anonymous).

Some participants perceived vaccines to be a low priority for MIH-CP because EMTs could provide the same service. One participant stated, “*I guess when I think community paramedicine*,* I think more of an advanced scope than vaccine distribution. Um*,* and here in the state of Indiana*,* EMT Basics are eligible to distribute vaccinations.*” (Non-MIH-CP-11). However, one of the state administrators clarified that EMTs can “*do influenza and COVID. We added that to their scope of practice. Anything else would have to be a paramedic for vaccination.”* (SRA-21).

### Vaccine program opportunities

Despite the challenges, many participants felt there were benefits to providing vaccines through MIH-CP and that their programs were successful. Many people viewed vaccines as “*beneficial*” (MIH-CP-01) and that MIH-CP could be an important part of reducing health disparities saying, “*I think that leans into a large ability to see the patient as a whole. And certainly*,* vaccines are within that ability to go into the home and do and make sure that everybody has equal access.*” (Non-MIH-CP-18). Agencies that had COVID-19 programs reported that they were successful. One said that, “*we ended up doing hundreds of vaccines. I can’t remember how many*,* but it was a lot of them*.” (MIH-CP-10). Another described the response to their services as:

“Oh, incredibly successful. You know, the whole concept was it wasn’t just the clinics that were successful. I say clinic and that’s kind of a broad term ….And so part of these clinics were us going to these individuals homes and giving them these vaccinations on site in their own homes. And that part of this was just, you know, I thought, wildly successful too. Because, you know, here we are taking care to people who otherwise wouldn’t have a means of getting there. And I think that that’s the kind of health care system we need to start moving towards in a lot of respects, not just in vaccinations.” (MIH-CP-07).

## Discussion

In this qualitative study examining implementation of MIH-CP vaccination programs, participants reported a wide variety of vaccination program structure and functions. Overall, vaccine programs were described as very successful and have the potential to serve as an effective way to improve access to underserved areas. The largest overall challenge reported was funding for the program, and the lack of funding had a ripple effect, affecting multiple functions within the organization, resulting in a lack of dedicated staff for vaccines and a perception that vaccinations were a low priority for the organization. Some participants commented on upstream causes of the lack of funding, including that there are not state-wide and federal policies governing MIH-CP, which limits reimbursement opportunities and limits the implementation of broader vaccination programs. Most participants described their vaccination programs as very successful and a way to reach people who were homebound or otherwise unable to access vaccines within their communities. The overall sentiment was that while vaccine hesitancy was not a barrier with the patient population they were serving, they did express discomfort at the prospect of being perceived as “pushing” or advocating for vaccines.

When discussing the feasibility of implementing an MIH-CP vaccine program, the main barrier described was funding. This was described as a barrier at multiple levels, including gaining initial funding, maintaining funding, and having dedicated staffing when sustained funding is not guaranteed. This same barrier has been reported in the literature for community health workers (CHWs), with one study also conducted in Indiana specifically reporting on the difficulties maintaining personnel with uncertain funding mechanisms and a cumbersome and confusing structure to apply for Medicaid reimbursement [29]. Like our findings, the CHW study reported that inconsistent funding jeopardizes CHW programs and recommended clarifying the existing Medicaid reimbursement policies. Recently, Indiana county health departments have received an influx of public health funding from the state that increased funding for public health in the state by 1500% [30]. Some of these funds are being used to expand the geographic reach of existing MIH-CP programs. This increased funding should alleviate the barriers discussed by our participants. Future work should examine the effects of this funding on alleviating disparities in the expanded areas.

Overall, CP vaccination programs were perceived as acceptable across EMS organizations. Our participants also reported they believed community members would be supportive of receiving vaccines from a CP. However, there have been limited studies examining patient perceptions of the acceptability of CP vaccine provision, particularly in the U.S [2]. Similar studies examining patient acceptability of the CHW-model has shown overall positive perceptions and high acceptability both in the U.S [31], and abroad [32, 33]. Future studies should examine community perceptions of CP acceptability to determine whether this might be a model that could be implemented more broadly to address health disparities.

While CPs in our study did report they felt comfortable giving vaccines, most also expressed that they would not want to advocate for vaccines or be seen as “pushing” vaccines on their patients. Even though they did not report any personal vaccination hesitancy in the qualitative interviews, the answers on the anonymous survey did indicate a significant level of vaccination hesitancy in this group of providers. This sentiment is seen across health professionals with one publication finding that nearly one-third of US healthcare providers were hesitant about vaccinations [34]. This is not a new phenomenon and vaccine hesitant providers existed before the COVID-19 pandemic and continue to exist after the pandemic [35]. Thus, there is a pressing need not only to educate healthcare providers to reduce vaccination hesitancy among this group, but also to give providers across the spectrum adequate training to effectively communicate with patients so that they feel comfortable combatting existing misinformation to improve vaccination uptake.

This study is among the first to examine feasibility and acceptability of implementing vaccination programs within MIH-CP programs. The findings can be used to inform implementation of other programs and to improve existing programs. However, the results should be interpreted in light of several limitations. First, the participants in this study are from a single state and the findings may be different in other geographic locations. Second, there were counties within the state that had no EMS services, and we were not able to gain perspectives of professionals working in those counties. Third, while the qualitative nature of our study allowed us to gain an in-depth understanding of the existing programs, we did not have quantitative data assessing program effectiveness and we are unable to determine if the implemented programs have had an impact on the health of the community.

## Conclusions

This study provides important context on the feasibility and acceptability of implementing an MIH-CP vaccination program. Major barriers to implementing and maintaining these programs are lack of sustained funding and unclear policies governing the programs. While participants in our study did not describe vaccine hesitancy as a major problem in their communities, they also expressed discomfort in advocating for vaccines, should people express hesitancy. They also described vaccines as a lower priority for their agencies than other services they provide, like managing chronic diseases. However, many did describe vaccines as beneficial and an important part of reducing health disparities in their communities. Future research should conduct rigorous evaluations of MIH-CP programs to determine program effectiveness and examine patient perceptions of the acceptability of receiving a vaccine from a CP. Using CP to deliver vaccinations to underserved communities has the potential to reduce health disparities and improve health outcomes for these communities.

## Electronic supplementary material

Below is the link to the electronic supplementary material.

Supplementary Material 1

Supplementary Material 2

Supplementary Material 3

Supplementary Material 4

Supplementary Material 5

## Data Availability

The datasets used and/or analyzed during the current study are available from the corresponding author on reasonable request.

## Abbreviations

CHW: Community health worker
CP: Community paramedics
DHS: Department of Homeland Security
EMS: Emergency Medical Services
MIH-CP: Mobile integrated health-community paramedicine
RHC: Rural health clinics
SES: Socioeconomic Status
SRA: State/regional administrators
