# Supplementary material for: Exploring the feasibility and acceptability of community paramedicine programs in achieving vaccination equity: a qualitative study
Source: BMC Health Serv Res. 2024 Sep 4;24:1022. doi: 10.1186/s12913-024-11422-0 (PMC11375945; doi:10.1186/s12913-024-11422-0)
Supplement: Supplementary file 1 — Supplementary Material 1 [file 12913_2024_11422_MOESM1_ESM.pdf]

Interview Guide

For Paramedicine Agency Leadership

1. To get us started, could you just briefly describe your current role with [Paramedicine Agency] and EMS?

   Probes:
   - About how long have you been in this role?
   - What kinds of paramedicine-related roles did you do prior to this role?

2. Could you tell me a bit about your agency?

   Probes:
   - Are you a municipal or private service?
   - About how many EMTs are employed? Paramedics? Others?
   - How would you describe the community you serve?

3. Do you currently have any community paramedicine programs?

   Probes:
   - If no: has your agency had any programs in the past?
   - If yes: what types of community paramedicine programs do you currently run?

   Probes:
   - What counties are served by your programs?
     - If multiple counties: Are all your programs available in all counties?
   - What was your first program? When was it started?
   - (If applicable) When were the other programs started?
   - What types of health outcomes are you trying to improve with your programs?
   - Do any of your programs provide vaccines?
     - If multiple counties above: Where are your vaccine programs available?

(Current/Past MIH-CP but not Vaccine Program)

Now I'd like to ask you some questions about your current community paramedicine program.

1. Could you walk me through the process your department went through from first hearing about community paramedicine to implementing your first program?

   Probes:
   - How did you first hear about community paramedicine?
   - Who developed your community paramedicine programs?
   - Who made the decision to implement the program?
   - What kind of proof or evidence that the program worked did you need to decide to implement the program?
   - How did you know what kind of community paramedicine program to choose?
   - Did you pilot the program before full-scale implementation?

2. Do you think your interventions have been effective?

   Probe:
   - What changes did you see that indicated to you that the interventions were effective?
     - If yes to success: What kinds of things do you think helped your program be successful?

3. What kinds of barriers did you find when starting to implement community paramedicine programs?

   Probes:

- Funding?
- Community accepting the program (follow-up if this is a barrier: What do you think about the community's concerns about the program?)?
- Spreading the word?
- Paramedic interest?

4. Looking back, what would you have done differently when starting your programs?
    - What resources would have been helpful when you were starting your program? In other words, was there anything you needed that would've made things easier to get the program started?

5. How are your programs funded?

6. (Only past programs): What caused your program to end?

(Current/Past MIH-CP with Current/Past Vaccine Program)

We're interested in knowing more about your vaccine program, specifically.

1. Could you walk me through the process your department went through from first hearing about community paramedicine to implementing your vaccine program?

Probes:

- How did you first hear about community paramedicine?
- Who developed your community paramedicine programs?
- Who made the decision to implement the program?
- What kind of proof or evidence that the program worked did you need to decide to implement the program?

2. Thinking back to when you implemented your vaccine program, why did you decide to implement it?

3. How did you know what kind of community paramedicine program to use?

4. Was there a champion for the program? That is, someone who really thought the program was important?
    - If yes— who served as the champion and how did they convince others to want to launch the program?

5. Was the program based on someone else's program or did you create it from scratch?
    - If someone else—What made you decide to model after their program? EX: Evidence that their program works, strong connections to other program, leadership liked the program.
    - If scratch—How did you decide what to include in the program?

6. Was the program piloted before you went to full-scale implementation?

7. Talk me through some of the logistics of running a vaccine program.
    Probes:
    - Acquire vaccine?
    - Standing order from local doc or just medical director?
    - Vaccine storage?
    - Administration?
    - Training?
    - CHIRP use?

- Buy-in from the paramedics?

8. Overall, how much does it cost to run your program, including things like acquiring the vaccines, advertising, and community paramedic time?
    - Are there other costs that I didn't mention?
    - Are you able to bill or be reimbursed for the vaccines?

9. What kind of local or state policies or regulations influenced the way that you implemented the program?

10. When you started talking about this kind of program, how did people within the agency, like paramedics or your medical director, respond?

Probe to determine if this is a personally held belief:

- What do you think of their positive/negative response?

11. What kinds of challenges did you experience when launching this program?

Probes:

- What concerns did your paramedics (the ones delivering the vaccines) have?
- What concerns did you personally have?
- How did they feel about offering vaccines to the community?

12. If you were going to help someone else implement this program, what would they need to know?

Probe:

- What parts of the program do you think are absolutely critical?
- How much funding would they need for the first year?
- What kind of challenges should they expect?
- What kind of training would be needed for the paramedics?

13. How did the community respond to this vaccine program?
    Probes:
    - How do you advertise or promote vaccines with your community members?
    - How do your paramedics navigate vaccine recommendations, addressing vaccine questions and vaccine hesitancy, etc?

14. Do you think your program has reached its goals?
    Probes:
    - How do you know?
    - What metrics do you use to evaluate the program?

15. Is there anything else that you think we should know about your vaccine program or community paramedicine programs more broadly?

16. (Only past programs): What caused your program to end?

17. Is there anything else that we didn't ask about, but you think would be important for us to understand?

(Without Current CP and Vaccine Program)

We'd like to know more about your opinions regarding community paramedicine programs in general.

1. What types of community paramedicine programs have you heard about?

Probes:

- How did you hear about them?

2. Are there non-emergency programs that your department runs? This might be things like car seat installations or smoke detectors checks.

3. (If yes to non-emergency programs) Could you walk me through the process your department went through from first hearing about [name the program] to making it happen?

Probes:

- How did you first hear about this type of program?
- Who developed the program?
- Who made the decision to implement the program?
- What kind of proof or evidence that the program worked did you need to decide to implement the program?
- How did you know what kind of program to choose?

4. If your department decided to implement a community paramedicine program, could you walk me through what that process would look like?

Probes:

- What type of health issue/outcome would you focus on? How would you decide that?
- Who would make the decision to implement the program?

5. What kind of proof or evidence that the program worked would you need to decide to implement the program?

6. What have you heard from others about implementing a community paramedicine program?

Probes:

- What things have gone well for other agencies? Not well?

(With/Without CP and without Vaccine Programs)

1. Have you heard of anyone using community paramedicine programs to distribute vaccines?

Probe:

- What are some of the things you've heard about these programs?
- What are your thoughts on that type of program being offered by paramedicine?
- What would you see as the pros and cons to paramedicine offering vaccination (to adults)?

(If no—briefly describe a couple of the programs in Indiana. E.g., "These community paramedicine programs train experienced paramedics to provide vaccinations in the community as a physician extender. This can happen at community outreach events, in schools, or at other community centers.")

2. Imagine if your agency was thinking about implementing a vaccine-specific community paramedicine program. What kinds of things do you think would influence that decision?

   Probes:

   - What concerns would your paramedics (the ones delivering the vaccines) have?
   - How would *they* feel about offering vaccines to the community?
   - What concerns would you personally have?
   - How would *you* feel about offering vaccines to the community?

3. What would be the biggest barrier to implementing this kind of program?

4. What would help an agency like yours get a program like this going?

5. How do you think your community would respond to this type of program?

Probe:
- Could you tell me a bit more about why you think so?

6. Are there other groups in your community doing similar work to distribute vaccines?

7. Do you think state EMS leadership would support this kind of program? What makes you say that?

8. Is there anything else that we didn't ask about, but you think would be important for us to understand?
