# Supplementary material for: Exploring the feasibility and acceptability of community paramedicine programs in achieving vaccination equity: a qualitative study
Source: BMC Health Serv Res. 2024 Sep 4;24:1022. doi: 10.1186/s12913-024-11422-0 (PMC11375945; doi:10.1186/s12913-024-11422-0)
Supplement: Supplementary file 2 — Supplementary Material 2 [file 12913_2024_11422_MOESM2_ESM.pdf]

Interview Guide

1. To get us started, could you just briefly describe your professional role?

Probes:

- About how long have you been in this role?
- How does your role relate to community paramedicine?
- What kinds of interactions do you have with MIH-CPs and MIH-CP agencies?
- What kinds of paramedicine-related roles did you do before this role?

2. Could you tell me a bit about your division or section within the Indiana Department of Homeland Security?
   - What is their mission/role related to community paramedicine?

We've talked with people in departments across the state, so we've started to understand perspectives from within the departments. But, it would be helpful to understand your perspectives on history of MIH-CP in Indiana and the functioning of MIH-CP across the state as a whole.

1. When did you hear about MIH-CP for the first time?
   - What were your first impressions?

2. How did MIH-CP get started in Indiana?
   - Any champions?
   - Reasons for starting?

3. What kinds of MIH-CP programs are currently functioning across the state?
   - How are these programs funded?
   - What kind of longevity do programs have? That is, do programs tend to start and keep going for many years, end in a few years because of funding or other challenges, or something else?

5. What are some of the goals of these programs?

   Probes:
   - Do you think the programs are reaching their goals
   - How do you know?
   - What metrics do you use to evaluate success?

6. What are the strengths of the current programs? Limitations or gaps?
   a. Gap in southern Indiana

7. What kinds of barriers do you think exist when agencies are starting community paramedicine programs?

   Probes:
   - Funding?
   - Community accepting the program
   - Spreading the word?
   - Paramedic interest?
   - Staffing?

8. If you were going to advise someone else to implement this program, what would they need to know?

Probe:

- What parts of the program do you think are absolutely critical?
- How much funding would they need for the first year?

- What kind of challenges should they expect?
- What kind of training would be needed for the paramedics?
- What partnerships do you think are important for successful MIH-CP programs?
    - o What if key player is not receptive?

Now I'd like to ask you some questions about the state of Indiana's EMS oversight processes, specifically related to community paramedicine.

1. Could you walk me through the process a department has to go through to start a community paramedicine/MIH-CP program?
    - At what points are they required to interact with the state?
    - What kinds of things are departments required to have in place to start an MIH-CP program?
    - How is this the same or different from setting up a non-MIH-CP agency? Normal EMS agency?

2. Are there policies or regulations that govern how these programs function?
    - General agency policies or MIH-CP specific?
    - Where can we find these policies?

3. Looking back to when MIH-CP started, what would you have done differently when launching across the state?

4. What resources would have been helpful when these programs were started? In other words, was there anything you needed that would've made things easier to get the programs started?

5. Thinking about the administration and oversight of MIH-CP in the state, what's working well? What do you think could be going better?

The last few questions focus specifically on vaccine-related MIH-CP programs across the state

9. How many programs do vaccine administration/related work?
    a. Types of vaccines?
    b. When established?
    c. Perceptions of how received by community?

10. Who was the first to do MIH-CP vaccine administration in Indiana?
    a. Circumstances?
    b. Type of vaccine?

11. We've heard about the importance of state guidance/approvals related to the COVID vaccine. For example, some people have said that reimbursement for the cost of the vaccine or giving paramedics the ability to give vaccines have facilitated the process. Could you tell me a bit more about some of the policies that have facilitated or hindered giving vaccines?

    I've heard that there was some kind of approval that either gave paramedics the ability to give COVID vaccines or created a reimbursement process. I don't know much about it, but I've heard people talk about it as helping them launch COVID vaccine clinics/pop-ups.

12. Beyond COVID, what kind of local or state policies or regulations influenced vaccine administration by MIH-CPs?
    Probe(s):
    - How do Indiana's MIH-CP policies compare to other states that you're aware of? Does Indiana have more or less regulation?

- Are there state-level policies in other states that you think facilitate or hinder MIH-CP programs?

13. Talk me through some of the logistics of running a vaccine program.
    Probes:
    - Acquire vaccine?
    - Standing order from local doc, medical director, or state health commissioner?
    - Vaccine storage?
    - Administration?
    - Training?
    - Are they able to upload records to CHIRP? How frequently do they use CHIRP?
    - Buy-in from the paramedics?

14. Is there anything else that you think we should know about your vaccine program or community paramedicine programs more broadly?

15. Can you think of anyone else at the state that would be important for us to talk to? Are the EMS district managers involved with MIH?

16. Is there anything else that we didn't ask about, but you think would be important for us to understand?
