# Supplementary material for: Exploring the feasibility and acceptability of community paramedicine programs in achieving vaccination equity: a qualitative study
Source: BMC Health Serv Res. 2024 Sep 4;24:1022. doi: 10.1186/s12913-024-11422-0 (PMC11375945; doi:10.1186/s12913-024-11422-0)
Supplement: Supplementary file 3 — Supplementary Material 3 [file 12913_2024_11422_MOESM3_ESM.pdf]

Sect.	Construct	Item Text	Response Options
**BLOCK A: PARTICIPANT DEMOGRAPHICS**			
A.00	*Socio-demographic variables*	**The following questions are about you**	
A.01	*Agency*	What best describes your agency?	1= Private EMS<br>2= Hospital-based EMS<br>3=Government/Municipal EMS<br>4=Volunteer Ambulance/Fire<br>5= State Agency (e.g., DHS, FSSA)<br>6= Other (please specify)
A.02	*Tenure*	How long have you worked in Emergency Medical Services in any role? (years, months)	[Fill in the blank]
A.03	*Age*	What is your age? (years)	[Fill in the blank]
A.03	*Sex*	Are you:	1= Male<br>2= Female<br>3= Prefer not to answer
A.04	*Ethnicity*	Are you Hispanic or Latino/a?	1= Yes<br>2= No
A.05	*Race*	How do you describe your race? (mark all that apply)	1= White/Caucasian<br>2= Black/African-American<br>3= Asian<br>4= American Indian/Alaska Native<br>5= Native Hawaiian/Pacific Islander<br>6= Other (please specify)
A.06	*Vaccine Uptake*	Have you personally received any COVID-19 vaccine?	1= No, I have no received any COVID-19 vaccines<br>2= Yes, I have received one COVID-19 vaccine<br>3= Yes, I am fully vaccinated (that is, I received at least 2 doses of either Pfizer or Moderna vaccines or one dose of Johnson & Johnson)<br>4= Yes, I am fully vaccinated and also received at least one booster shot
**BLOCK B. VACCINE BELIEFS**			

B.00	*Vaccine Beliefs*	**Please indicate how strongly you agree or disagree with the following statements:**	
B.01	*Important*	COVID-19 vaccines are important for my health	1= Strongly disagree<br>2= Disagree<br>3= Neither disagree nor agree<br>4= Agree<br>5= Strongly agree
B.02	*Perceived Efficacy*	Getting a COVID-19 vaccine is a good way to protect me from coronavirus disease	1= Strongly disagree<br>2= Disagree<br>3= Neither disagree nor agree<br>4= Agree<br>5= Strongly agree
B.03	*Trust*	Any COVID-19 vaccine approved by the FDA and recommended by the CDC is effective	1= Strongly disagree<br>2= Disagree<br>3= Neither disagree nor agree<br>4= Agree<br>5= Strongly agree
B.04	*Altruism*	Getting a COVID-19 vaccine is important for the health of others in my community	1= Strongly disagree<br>2= Disagree<br>3= Neither disagree nor agree<br>4= Agree<br>5= Strongly agree
B.05	*Benefits*	A COVID-19 vaccine is beneficial to me	1= Strongly disagree<br>2= Disagree<br>3= Neither disagree nor agree<br>4= Agree<br>5= Strongly agree
B.06	*Recommendation*	I do what my doctor or health care provider recommends about a COVID-19 vaccine	1= Strongly disagree<br>2= Disagree<br>3= Neither disagree nor agree<br>4= Agree<br>5= Strongly agree
B.07	*HCP Trust*	The information I receive about COVID-19 vaccines from my health care provider is reliable and trustworthy	1= Strongly disagree<br>2= Disagree<br>3= Neither disagree nor agree

			4= Agree<br>5= Strongly agree
B.08	*CDC Trust*	The CDC provides trustworthy information on COVID-19 vaccines	1= Strongly disagree<br>2= Disagree<br>3= Neither disagree nor agree<br>4= Agree<br>5= Strongly agree
B.09	*Organizational Trust*	I trust COVID-19 vaccines because medical organizations recommend them	1= Strongly disagree<br>2= Disagree<br>3= Neither disagree nor agree<br>4= Agree<br>5= Strongly agree
B.10	*Safety*	COVID-19 vaccines have not been around long enough to be sure they are safe	1= Strongly disagree<br>2= Disagree<br>3= Neither disagree nor agree<br>4= Agree<br>5= Strongly agree
B.11	*Side Effects*	I am concerned about serious side effects of COVID-19 vaccines	1= Strongly disagree<br>2= Disagree<br>3= Neither disagree nor agree<br>4= Agree<br>5= Strongly agree
B.12	*Long-term Effects*	I think COVID-19 vaccines might cause lasting health problems for me	1= Strongly disagree<br>2= Disagree<br>3= Neither disagree nor agree<br>4= Agree<br>5= Strongly agree
**BLOCK C. QUESTIONS/COMMENTS**			
C.00	*Conclusion*		
C.01	*Other*	Do you have any other questions or comments you think we should know as we move forward with this project?	[Open-ended response]
