# Supplementary material for: Exploring the feasibility and acceptability of community paramedicine programs in achieving vaccination equity: a qualitative study
Source: BMC Health Serv Res. 2024 Sep 4;24:1022. doi: 10.1186/s12913-024-11422-0 (PMC11375945; doi:10.1186/s12913-024-11422-0)
Supplement: Supplementary file 4 — Supplementary Material 4 [file 12913_2024_11422_MOESM4_ESM.pdf]

Code System

Code System
**Has Distributed Vaccines**
Yes
No
**Types of Vaccines**
Hep A
Childhood vaccines
Tetanus
Anything provided by the health department
Flu
COVID
Anything ordered by the provider
**Successes/Strengths**
No concerns
Beneficial for community
Respondent perceives success
**Barriers/Challenges**
Better fit for other services
Concerns about funding
Vaccine hesitancy
Observed hesitancy
People requested vaccines
Other
None from community
Policies/need for policies
**Administration**
Documentation
Other
Primary care

Health department
Internal
Training/background
Role/possible role of EMTs
Storage of vaccines
**Structures**
Standalone program
Extension of current services
Outreach of another program
**Funding**
Current Approaches
Grants
Donations
Existing budget
Fee for service/reimbursement/billing
